# Using a geographic information system to enhance patient access to point-of-care diagnostics in a limited-resource setting

**DOI:** 10.1186/s12942-016-0037-9

**Published:** 2016-03-01

**Authors:** William J. Ferguson, Karen Kemp, Gerald Kost

**Affiliations:** UC Davis Point of Care Testing Center for Teaching and Research, School of Medicine, University of California, Davis, 3488 Tupper Hall, Davis, CA 95616 USA; Spatial Sciences Institute, Dana and Davis Dornsife College of Letters, Arts, and Sciences, University of Southern California, Los Angeles, CA USA

**Keywords:** Acute myocardial infarction, Spatial care path™, Health care access, Geographic information system (GIS)

## Abstract

**Background:**

Rapid and accurate diagnosis drives evidence-based care in health. Point-of-care testing (POCT) aids diagnosis by bringing advanced technologies closer to patients. Health small-world networks are constrained by natural connectivity in the interactions between geography of resources and social forces. Using a geographic information system (GIS) we can understand how populations utilize their health networks, visualize their inefficiencies, and compare alternatives.

**Methods:**

This project focuses on cardiac care resource in rural Isaan, Thailand. A health care access analysis was created using ArcGIS Network Analyst 10.1 from data representing aggregated population, roads, health resource facilities, and diagnostic technologies. The analysis quantified cardiac health care access and identified ways to improve it using both widespread and resource-limited strategies.

**Results:**

Results indicated that having diagnostic technologies closer to populations streamlines critical care paths. GIS allowed us to compare the effectiveness of the implementation strategies and put into perspective the benefits of adopting rapid POCT within health networks.

**Conclusions:**

Geospatial analyses derive high impact by improving alternative diagnostic placement strategies in limited-resource settings and by revealing deficiencies in health care access pathways. Additionally, the GIS provides a platform for comparing relative costs, assessing benefits, and improving outcomes. This approach can be implemented effectively by health ministries seeking to enhance cardiac care despite limited resources.

## Background

We developed a geographic information system (GIS) as a framework for (a) understanding how populations utilize a health small-world network in a limited-resource setting, (b) quantifying population access to health resources, (c) visualizing inefficiencies, and (d) evaluated alternative scenarios that build on a wide array of previous applications [1–7]. Prior studies are limited because they do not focus on social pressures of a health system, such as culturally related health decisions, or how diagnostic information is obtained.

The complexities of health networks are not explained by the sum of their parts and often are defined by the natural connectivity that arises from their element interactions [8]. Health networks are thought of as small-world networks (SWN), which are not completely random or regularly connected systems [9], and may not be efficient in terms of the urgent care of its populations. The goal of this study, therefore, is to show that a GIS can help understand the inefficiencies that exist within health network SWNs, and then, used to enhance strategic organization and outcomes.

Point-of-care technologies (POCT) result from the miniaturization of conventional laboratory diagnostic tests into portable forms. Many POCT are characterized by factors such as, affordable, sensitive, specific, user-friendly, rapid/robust, equipment-free, and deliverable (ASSURED) criteria [10]. ASSURED qualities allow POCT to be used in non-laboratory settings by non-technical staff without infrastructure, such as in-wall electricity, and in a wide range of environments. POCT has been implemented in national disaster caches for disaster preparedness [11], emergency settings for care optimization [12], and limited-resource settings [13, 14].

Kost et al. [15] attempted to quantify SWN relationships for cardiac support in health facilities in Northeastern Isaan, Thailand. Their study revealed isolated regions with inadequate support for their populations. They suggested several placement strategies for POCT that would improve patient outcomes. While the study was useful for understanding the SWN, it did not attempt to quantify population access to these diagnostic resources nor evaluate how the recommended placement strategies would affect this access.

GIS provides the ability to effectively implement POCT in health networks to streamline decision making at the point of care. This can (a) improve patient outcomes, (b) save resources including money and time, and (c) ensure that the health networks are sufficiently robust for a disaster or emergency event. This project demonstrates how a GIS can quantify health care access to help make decisions on how to integrate POCT.

The goal of this project is to create, evaluate, and utilize a spatial analysis to improve health networks by quantifying pathways of individuals towards a diagnosis and then care. This analysis was evaluated within the study area used in the Kost et al. [15] study focusing on the diagnosis and care of individuals with acute myocardial infarction (heart disease). The goals are:

- To build and evaluate a spatial analysis that defines health care access.
- Define the current health care access to cardiac care within an area used in the Kost et al. [15] study.
- Evaluate implementation strategies that improve health care access to cardiac care.
- Determine the outcomes of the implementation strategies against current access.

## Results

### Current health care access for cardiac events

The results of the current health care access analysis are summarized in Table 1 split into two access components: (a) populated places to diagnosis and (b) diagnosis to care. The mean travel time from all populated places to care of 219 min (3.6 h) is dominated by the mean travel time from diagnosis to care of 169 min (2.8 h). The minimum total travel time from any populated place was 1.6 h and the maximum was 5.5 h, a difference of 3.9 h.

**Table 1:** Mean (SD) travel time for current health access

Health access	Mean (SD) Travel time (min)
Populated places to diagnosis	49.9 (25.5)
Diagnosis to care	168.6 (37.1)
Populated places to care	218.5 (39.5)

**Fig. 1:**
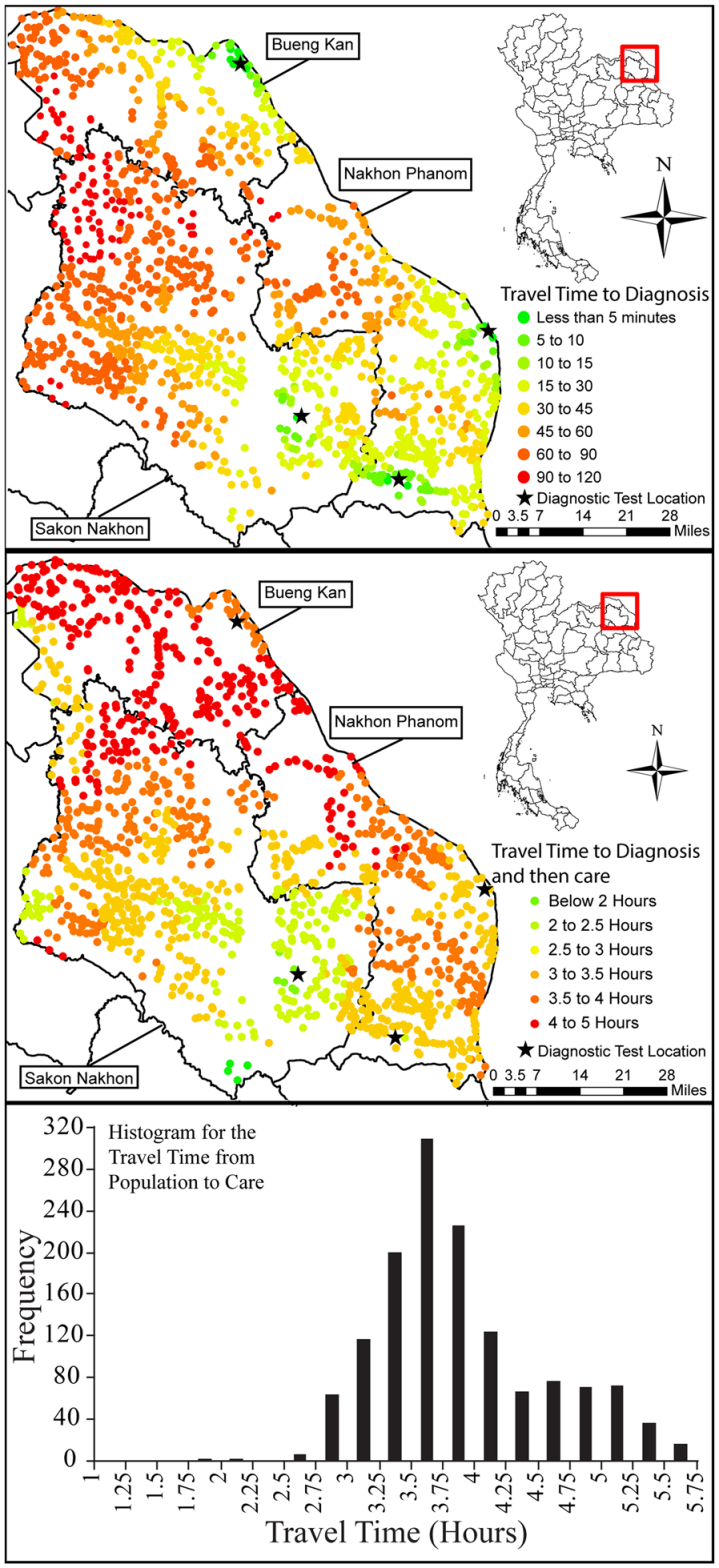
Travel times to diagnosis (*top frame*) and care (*middle frame*) for current health care access. Histogram (*bottom frame*) shows distribution of travel time to care in 15 min bins

Travel time from populated places to diagnosis is shown in Fig. 1 (top frame). Lower travel times occur in the eastern parts of the provinces, nearer to the diagnostic facilities. Concentrations of higher travel times occur in the north-western areas of Sakhon Nakhon and western areas of Bueng Kan where individuals must travel over 1.5 h to reach a diagnosis.

Figure 1 (middle frame) shows the total travel time from populated places to diagnosis then to care. In the western areas of Bueng Kan there are groupings of populated places that have under 2 h to reach care. Immediately surrounding these locations, travel time of these populated places increases to between 5 and 6 h. Individuals who travel east must then backtrack to reach care. A longer time to diagnosis in order to decrease overall time to care may lead to a more appropriate evaluation.

The longest travel times to care mostly fall in Bueng Kan. A small area of higher travel times can also be found in northern Sakhon Nakhon which may have resulted from individuals from this area travelling 1.5–2 h to reach a diagnosis and then backtrack to reach care. The locations further away from Srinagarind Hospital, where you would expect longer travel times, particularly eastern Nakhon Phanom, have relatively lower travel times.

Figure 1 (bottom frame) shows the histogram for the travel times from populated places to care and provides a means to understand the distribution of calculated routes.

### Widespread implementation strategy

Mean travel time for the two widespread implementation strategies as well as the current access times are shown in Table 2. Both widespread implementation strategies offer greatly decreased travel time when compared to the current diagnostic resource access.

**Table 2:** Mean (SD) travel time comparison between different policy implementation strategies

Health access	Current access	All hospitals	All health resource facilities
Average travel time from pop to diagnosis (min)	49.9 (25.5)	13.9 (7.4)	4.6 (3.5)
Percent decrease over current access	N/A	72.1 %	90.8 %
Average travel time from diagnosis to care (min)	168.6 (37.1)	155.9 (37.9)	159.4 (37.9)
Percent decrease over current access	N/A	7.5 %	5.5 %
Total travel time (min)	218.5 (39.5)	169.8 (39.9)	164 (38.0)
Mean travel time percent decrease over current access	N/A	22.3 %	24.9 %

The quickest routes are generated when POCT is implemented in every health resource facility; however it offers little advantage over integrating POCT in every hospital. The difference in average travel times from populated places to diagnosis between the two strategies was 9.3 min which is negligible when compared to the travel time from populated places to care. The difference between these two strategies would only result in a 2.6 % decrease, potentially a reason to only implement POCT within hospitals instead of every health resource facility.

**Fig. 2:**
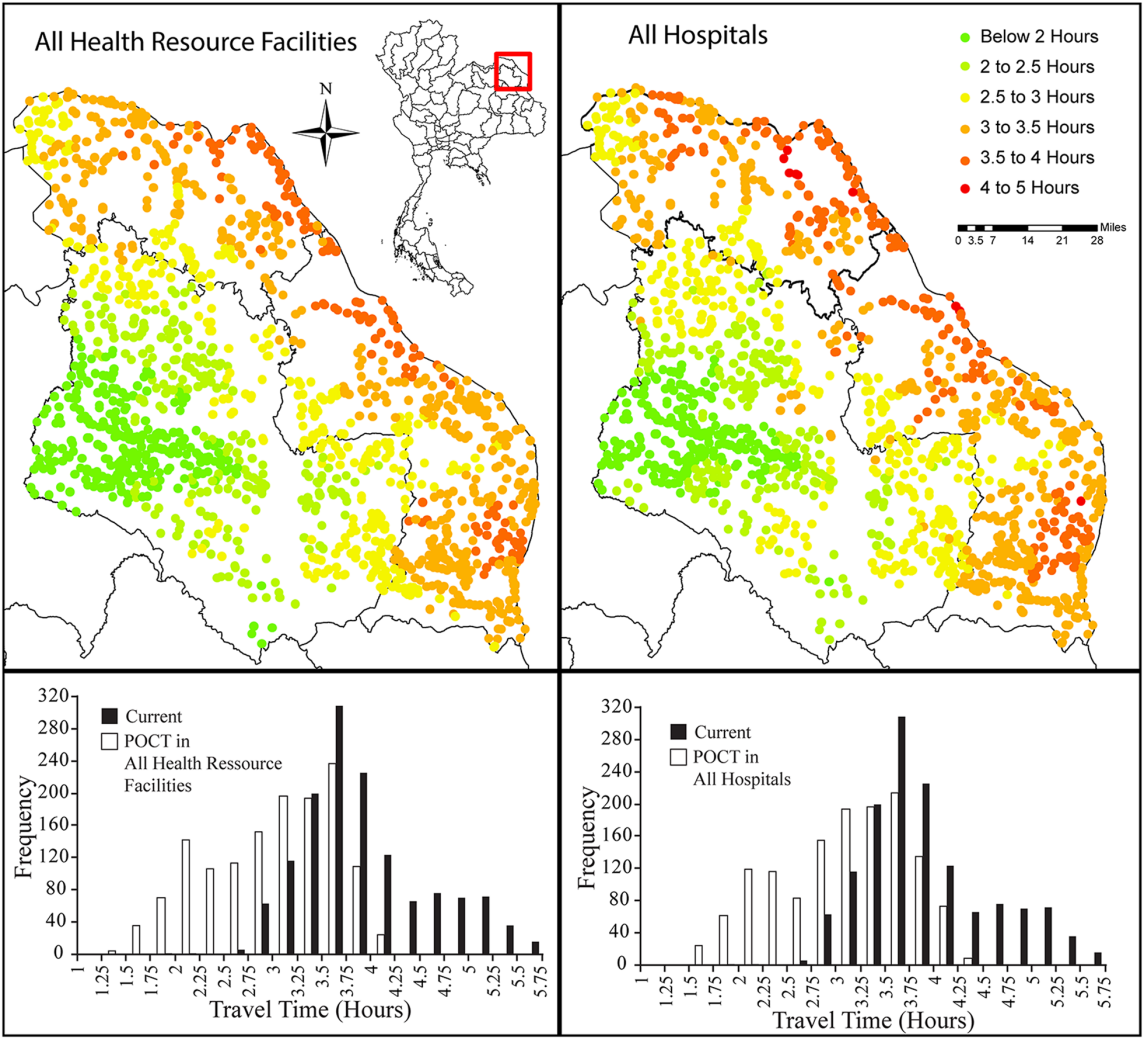
Comparison of widespread integration strategies where POCT is placed in all health resource facilities (*left frames*) and only in hospitals (*right frames*). While both strategies indicate improved access when compared with current health care access, neither offer improvements over the other

Figure 2 displays the results for the two strategies. In both cases, health care access travel time is lowest in western Sakhon Nakhon with travel times increasing smoothly outward. This reflects the fact that populated places further away from their eventual destination of Srinagarind Hospital must to travel longer to reach the hospital. This produces a more smoothly varying distribution of times when compared to the current health care access distribution where high travel times exist in small pockets or greatly increase over short distances. The two strategies generate the same visual results which suggest they would produce little differences in outcomes for individuals.

Histograms in Fig. 2 show the travel time to care distributions for the two widespread implementation strategies (white bars) compared to the current health care access (black bars). In both cases we see the improvements in overall travel times.

### Limited-resource implementation strategy

**Fig. 3:**
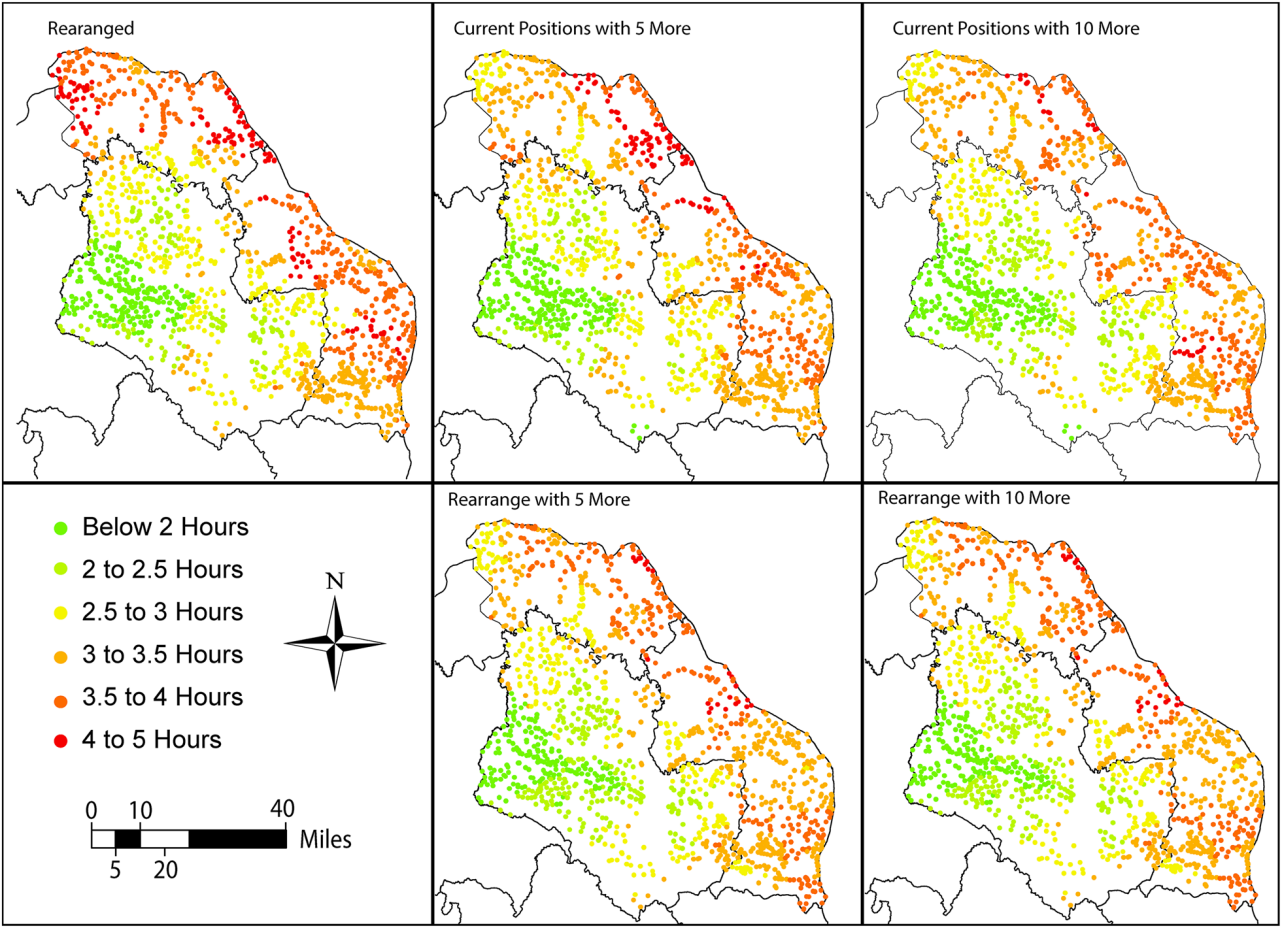
Travel time visual comparison between different low-resource implementation strategies

**Fig. 4:**
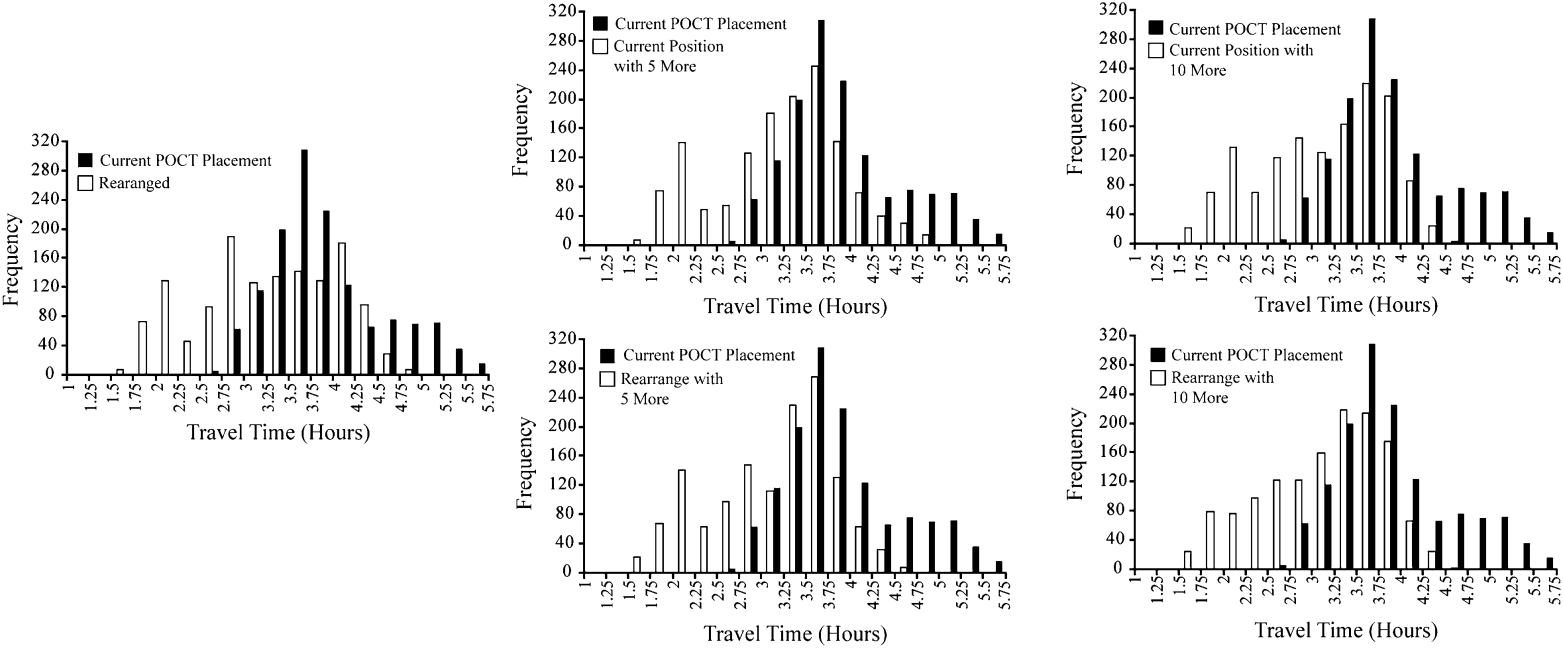
Histogram comparison between different low-resource implementation strategies

Table 3 details the results of the limited-resource analysis. When compared to current access, all strategies offered improvements, with a steady decrease in travel time as more POCT are available to be integrated into the health network. Figures 3 and 4 demonstrate the visualized improvements and corresponding histograms for the limited-resource implementation strategies.

**Table 3:** Travel time comparison between different low-resource implementation strategies

Health access	Current	Rearrange	Current position with 5 more	Current position with 10 more	Rearrange with 5 more	Rearrange with 10 more
Mean (SD) travel from population to diagnosis	49.9 (25.5)	30.6 (15.3)	26.8 (13.1)	21.8 (11.3)	23.3 (12.0)	19.6 (10.6)
Decrease compared to current	N/A	38.7 %	46.3 %	56.3 %	53.3 %	60.7 %
Mean (SD) travel from diagnosis to care	168.6 (37.1)	150.4 (41.4)	151.4 (39.5)	151.9 (39.9)	149.6 (37.8)	153.3 (38.1)
Decrease compared to current	N/A	10.8 %	10.2 %	9.9 %	11.3 %	9.1 %
Mean (SD) travel time from population to care	218.5 (39.5)	181 (46.1)	178.2 (42.7)	173.7 (41.9)	172.8 (40.6)	172.9 (39.8)
Decrease compared to current	N/A	16.7 %	18.4 %	20.5 %	20.9 %	20.9 %

The first analysis rearranges existing POCT resources to better support the population in the study area, and resulted in a decrease of travel time of 16.7 %. The second and third analyses involve keeping the current POCT where they are and adding an additional five and ten POCT, optimally located, to the health network. These analyses decreased travel time from populated places to care by 18.4 and 20.5 % respectively. It is worth noting that comparing rearranging existing resources to adding 5 additional POCT only results in a decrease of 1.7 %, suggesting little benefit.

In the last two analyses, rearranging existing resources while adding five and ten POCT, the overall effect on health care access is the same. The differences being a decrease to diagnosis by 3.7 with an increase to care by while the addition of the second additional five POCT decreases the time to diagnosis by 3.7 min negated by and increase to care of the same. Thus, it could be concluded that adding the five extra tests does not warrant the costs of adopting them, since overall it does not add any benefit.

## Discussion

This study successfully demonstrated that a GIS helps understand the role of diagnostic technologies within a SWN in rural Thailand. Although preliminary in nature, this analysis could help make informed decisions to improve health networks worldwide. It is important, however, to recognize some important limitations of the spatial analysis.

### Inputs

Complete, accurate, and appropriate data is one of the most important and biggest challenges for this analysis. OSM data was used for both populated places and roads because no other data was available. While OSM data is often criticized for its incompletes and inaccuracy in developing countries like Thailand, it may still be the most appropriate dataset for this type of analysis due to its availability.

The purpose of the road data is to help understand the generalized travel patterns for the population. This can be done by using a wide range of data sets from different sources. Government data sets like the US topologicaly intergrated geographic encoding and referencing (TIGER) provide a standardized data source collected by the government, however lack the integration of travel times or may not be available in some countries. Additionally, commercial data sets like Tom Tom^®^, Google Maps^®^ and HERE^®^ can provide the necessary travel time data, but are costly. When available government or commercial data sets may present a viable option to replace OSM used in this analysis.

While road data may be obtained from government or commercial sources, information about where people may originate is a source of considerable potential error. In this study populated places from OSM were used as origin points for travel. However, it is acknowledged that this data source may not be the most appropriate because there is no means to judge the accuracy or completeness of this of dataset and the spatial scale of aggregation may not be appropriate for this analysis.

To overcome incompleteness and inaccuracy of OSM data, census data can be used if available. For this study, no census data of appropriate spatial scale was available. When available, census aggregations may provide a better and more standardized way of obtaining origins of travel. However, even when official census data is available, the collection timeframe and the scale of spatial aggregation may suffer to the same problems OSM data has.

If population data cannot be obtained through outside sources, then it must be collected. While this can be a time and resource intensive process whose cost may not justify the benefits, a recent study showed that low cost collections of village locations can be done easily and rapidly [18].

No matter what dataset is used in this analysis to represent populations or road networks, consideration for how this data effects the analysis must be considered. Having inappropriate data may cause the analysis to miss subsets populations who could benefit from increased access to health resources.

### Analysis

The analysis assumes that an individual chooses to take the quickest path to their. There are several different ways in which an individual may realistically divert from this assumption. First, a patient who self-identifies as critically ill may decide to travel in a more direct path knowing their ending destination will be Srinagarind Hospital. Second, a patient may not have the knowledge of or does not trust certain roads or health resource facilities and thus alters their path, from a more optimal one.

These diversions from the assumed paths represent the concept of SWN, where individual paths through the network directly reflect the cultural or social pressures that exist. A way to accurately include this into the analysis is to survey the population or health professionals to better understand their attitudes towards their health network and understanding the critical paths.

Another assumption is that the quickest path to diagnosis will lead to the quickest path to care. The analysis only optimizes the time to diagnosis. Traveling to the closest facility with appropriate diagnosis may not be the most effective overall care path. Thus, another way to pre-position supplies is to optimize travel from populated places such that travel to care is optimized.

While the analysis takes relatively little processing power or time, the software cost may be prohibitive for use by the rural health networks that would benefit the most. The open source community has software packages that can easily replicated the workflows used in this analysis. While the cost for the software is free the technical requirements are considerably higher to implement something similar. Future efforts will be done to implement an open source application that can be easily applied.

### Recommendations for the future

Health costs have risen consistently over the last few years. This increase is partly associated with technological developments. At the same time, integration of new technology is often slow, difficult, and expensive. Unfortunately, health networks may not have the means to understand the benefits of adopting technologies or to compare those benefits to the costs of adoption [19]. The research described here is the basis of what has become the spatial care path™ (SCP). The SCP is defined as the most efficient route available to individual patients within health networks, and evaluates health networks in a spatial context to improve decision-making and reduce costs [20].

In this analysis we applied SWN analysis in a simplified fashion. More advanced methods incorporating SWN dynamics and detailed location-allocation models could greatly improve this analysis. A recent study published uses a modified P-median model to understand where best to place new facilities within health networks [21]. The limitations of these models are that they focus on generalized health access instead of focusing on the SCP for individual diseases. The SCP analysis could be incorporated into these advanced methodologies to provide a unique perspective on the spatial benefits.

The SCP concept has been expanded to include other disease paths including diabetes management and the Ebola outbreak [22, 23]. Research is currently underway to understand how variations in data completeness, health network infrastructure, and topological variations impact the results of the analysis described here. This project will explore if a compelling argument can be made using this type of analysis to motivate the integration of POCT into health networks. One of the additional outcomes of this study is a discussion on the data sources used and their limitations, ideally motivating the creation of more appropriate datasets in the future. The hope for the SCP is that it will fit into future decision making for all health networks by supporting the evaluation of care paths in a spatial framework.

## Conclusions

Point of care technologies enables diagnostic decisions in new locations streamlining care paths, improving outcomes, and reducing inefficiencies. Spatial analysis estimated current mean travel time to cardiac care to be over 3.5 h. This could be reduced by as much as 25 % with integration of POCT, demonstrating the utility of a GIS to help make decisions. Although not without its limitations, this spatial analysis can help health networks (a) understand the benefit of integrating POCT within a rural health system, and (b) provide evidence of the potential benefits which can be used to justify the costs.

## Methods

This analysis uses a two-step method that defines health care access by quantifying how an individual would travel from (a) a place of origin to a location of diagnosis and then (b) from the diagnosis to a location of care. For this analysis travel is considered on road only as other travel medians (air or railway) are cost prohibitive or not available for the majority of the population. This process involves four data types: (a) populated places, (b) roads, (c) health resource facilities, and (d) cardiac diagnostic resources. Two implementation strategies were used to improve this health access. The first evaluated a widespread POCT integrations strategy; the second analyzed a limited resource strategy.

### Study area

A portion of the region of Isaan, Thailand, was used as the study area because it was previously investigated using paper-based surveys by the researchers in the Kost et al. [15] study. The Isaan region, often referred to simply as the Northeast region of the country, had 21,305,000 people in 2010 and is considered to contain the country’s most rural and poorest areas.

**Fig. 5:**
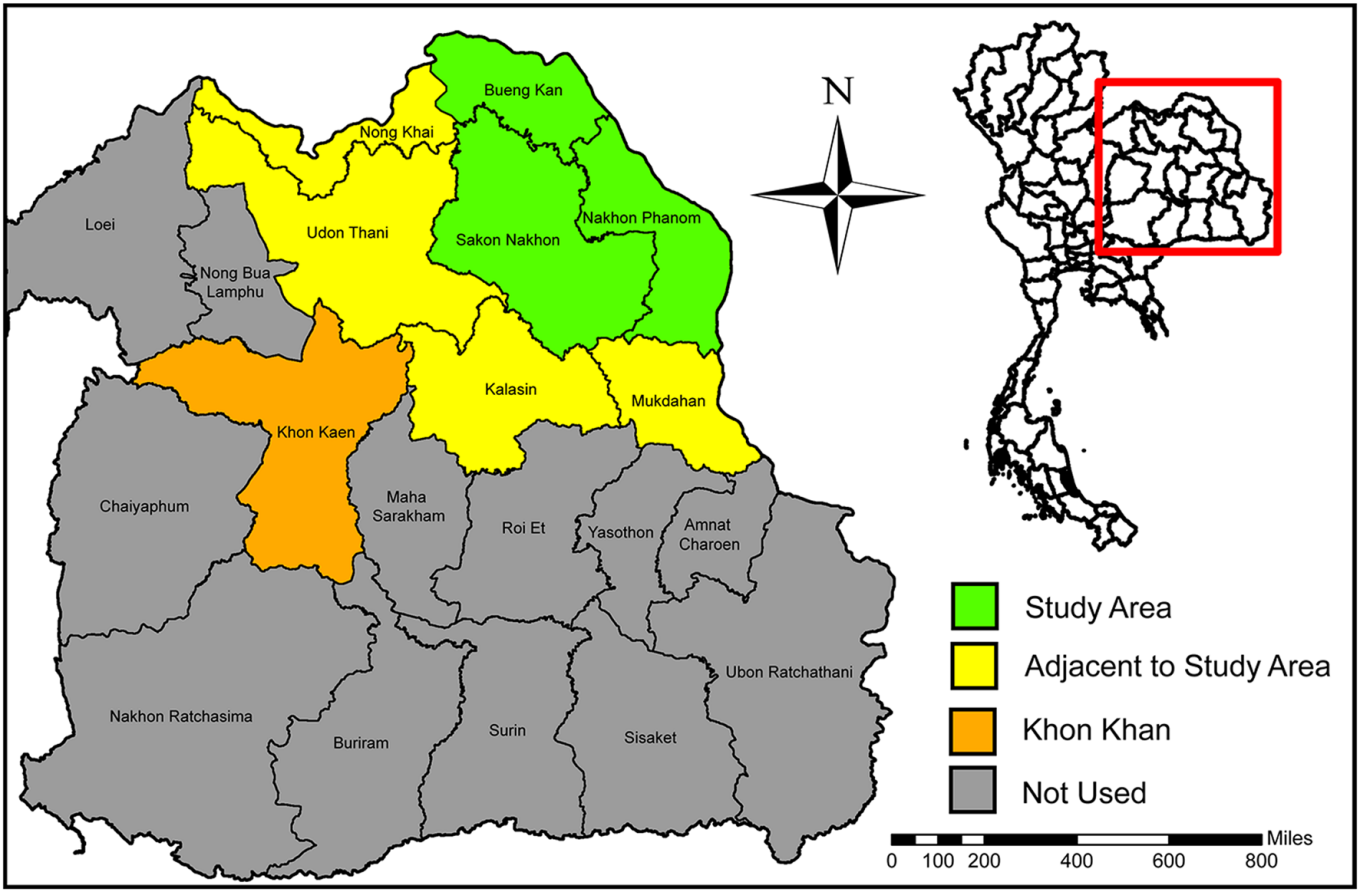
Location of the study area (*green*) used in this project as compared to the Isaan region. Khon Kaen (*orange*) contains Srinagarind Hospital, the available only cardiac care in the region. Therefore, patients originating from the study are must travel through the adjacent provinces (*yellow*)

Figure 5 shows the Isaan region consisting of twenty provinces, eight of which are used in this project. We will focus on the populations in the provinces of Bueng Kan, Sakhon Nakhon, and Nakhon Phanom. These three provinces are 5513 square miles in area and had a population of 583,726 in 2010. Bueng Kan came into existence in 2011 after it was separated from its neighbor Nong Khai [16]. The provinces of Nong Khai, Udon Thani, Kalasin, and Mukdahan border the main study area and are included because individuals must travel through these provinces to reach Khon Kaen. Khon Kaen province is the second largest province in Isaan and has one of the larger cities in the region containing Srinagarind Hospital, the only location of cardiac care in the region.

### Data

Table 4 provides an overview of the data and their sources. All data was projected to WGS 1984, UTM 47N, which is suitable for use for most of Southeast Asian countries including Thailand.

**Table 4:** Data sources

Data name	Theme/topic	Description	Date obtained
OpenStreetMap http://www.openstreetmap.org/	Roads and populated places	User submitted open data that emphasizes local knowledge. Contains both line and point data with attributes	Downloaded May 29th, 2014
MapMagic 2013: Thailand http://www.thinknet.co.th/	Health resource facilities	Proprietary point data collected and sold by a Thai based company	Assumed to represent the facilities that exist as of 2013
Global Administrative Areas http://www.gadm.org/	Thailand province	Lines representing political boundaries. Note: Bueng Kan was established in 2011 and the new boundary was added manually	Downloaded May 29th, 2014
Kost et al. [15]	Diagnostic technology locations	Resources for available cardiac diagnostic resource were surveyed in Isaan Region. Contains attributed point data	Collected throughout 2009 and 2010.

#### Population aggregations

**Fig. 6:**
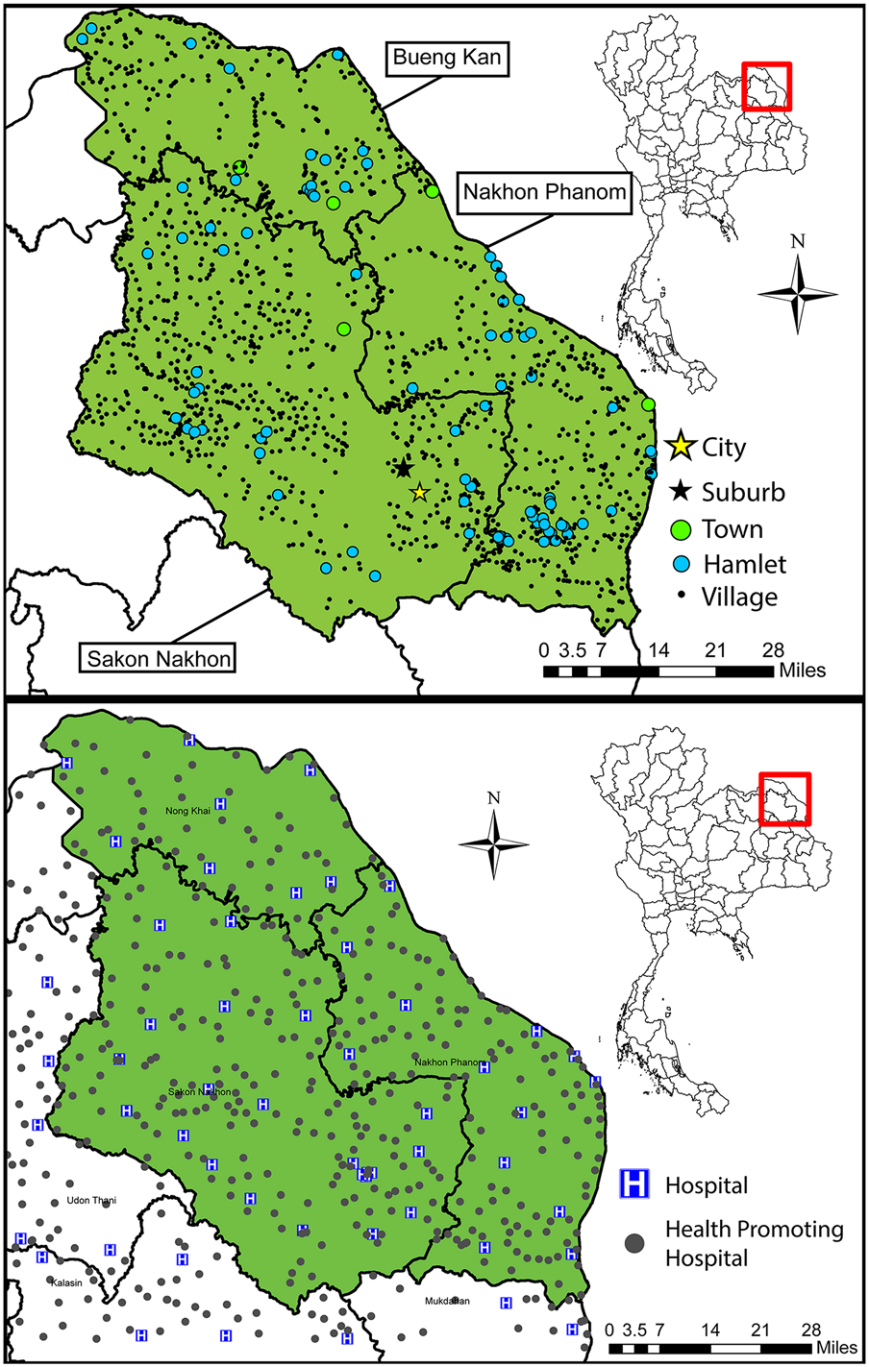
OpenStreetMap populated places (*top frame*) and MapMagic 13 health resource facility locations for the study are. Health resource facilities were included for the adjacent regions to account for edge effects

Since data about the distribution of population across the study area was not otherwise available, OpenStreetMap (OSM) data was used to represent where individuals may originate when they have a cardiac event. OSM uses points representing settlements such as cities, towns, villages, and suburbs. These are described according to the OSM metadata definitions in Table 5 along with estimated population totals. Population locations used in this study are illustrated in Fig. 6 (top frame). The population associated with these populated places amounts to 82 % of the total found in the region [16].

**Table 5:** OpenStreetMap populated place descriptions (descriptions modified from OpenStreetMap places metadata)

Type	Description	Population estimate	Count
City	Largest urban settlement in province which usually has more than 100,000 people	100,000	1
Suburb	A distinct section of an urban settlement with unknown population	100,000	1
Town	A second tier urban settlement of local importance with more than 10,000 people	20,000	5
Village	A smaller distinct settlement, smaller than a town with <10,000 people	10,000	1303
Hamlet	A smaller rural community with 100–200 people	200	83

#### Roads

Like population data, road data were also not readily available from official sources. Therefore, road data were also obtained from OSM. In the OSM data, there are over 26 different types of roads. Table 6 lists the road types used in this study area, their descriptions according to OSM, and estimated travel speed. Since these speeds are somewhat arbitrary, an in-depth sensitivity analysis of the effect of varying travel speed on health care access was performed during the development of the spatial analysis and reported elsewhere [17]. That analysis indicated that the speed estimates are sufficiently accurate for this study’s purpose.

**Table 6:** OpenStreetMap road descriptions

Highway type	OpenStreetMap description	Estimated speed (MPH)
Trunk	The most important roads in a country’s system that aren’t motorways	65
Primary	The next most important roads in a country’s system. (Often link larger towns.)	55
Secondary	The next most important roads in a country’s system. (Often link smaller towns and villages.)	45
Tertiary	The next most important roads in a country’s system	30
Unclassified	The least most important through roads in a country’s system—i.e. minor roads of a lower classification than tertiary, but which serve a purpose other than access to properties	25
Residential	Roads which are primarily lined with and serve as an access to housing	25
Service	For access roads to, or within an industrial estate, camp site, business park, car park etc	15
Living street	Residential streets where pedestrians have legal priority over cars, speeds are kept very low and children are allowed to play on the street	25
Road	A road where the mapper is unable to ascertain the classification from the information available	35
Connector roads	Roads added to the network to ensure connectivity between health facilities and population aggregation locations	35

#### Health resource facilities

Health resource facilities’ geographic coordinates were obtained from MapMagic 13, a product developed by THiNKNET (www.thinknet.co.th), a company based in Thailand. MapMagic 13 has two designations for health resource facilities: hospitals, which tend to be larger facilities with more robust diagnostic and care capabilities, and health promotion hospitals, which represent smaller hospitals and clinics. Provincial totals of health facilities are summarized in Table 7. Health resource facilities’ coordinates were manually recorded in degrees and loaded into ArcGIS as points.

**Table 7:** Health resource facility by province and type

Province	Bueng Khan	Sakhon Nakhon	Nakhon Phanom
Hospitals	8	23	13
Health promoting hospitals	51	171	125
Totals	59	194	138

#### Diagnostic technology locations

Data on the location of point-of-care technology within the Isaan region was collected by Kost et al. [15]. These locations may not represent current POCT status within the provinces; however the locations can be used to demonstrate the effects for the implementation strategies.

**Fig. 7:**
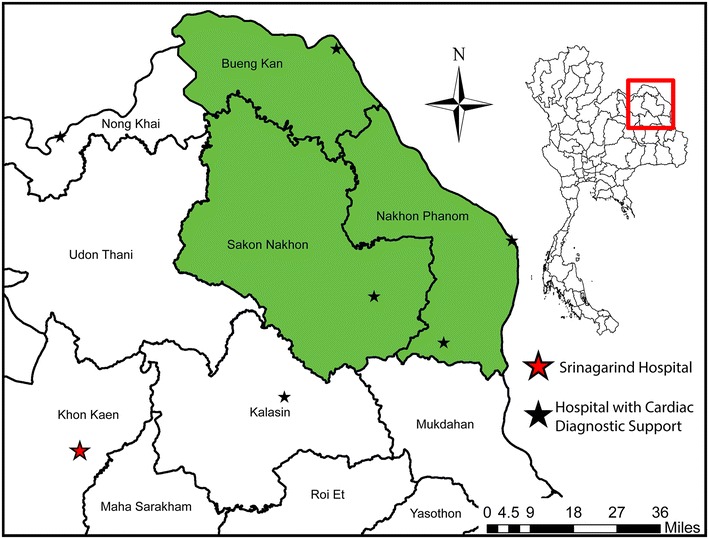
Health resource facilities with point-of-care resources are mapped as well as Srinagarind Hospital. Populations in the western portion of Sakon Nakhon or Bueng Kan must decide to travel east or west to reach cardiac diagnostic resources

Figure 6 (bottom frame) shows the locations of health resource facilities that could serve as a location for POCT. Facilities in the provinces adjacent to the study area were included as potential diagnostic sites to negate edge effects. Figure 7 shows the location of Srinagarind Hospital as well as the diagnostic resources identified.

### Methodology

Implementation of the spatial analysis required several stages: (a) creating a road network, (b) quantifying health care access under current conditions, (c) quantifying health care access under widespread integration strategies, and (d) using a location-allocation algorithm to select best implementation sites for new POCT locations under limited resource strategies.

#### Network creation

**Fig. 8:**
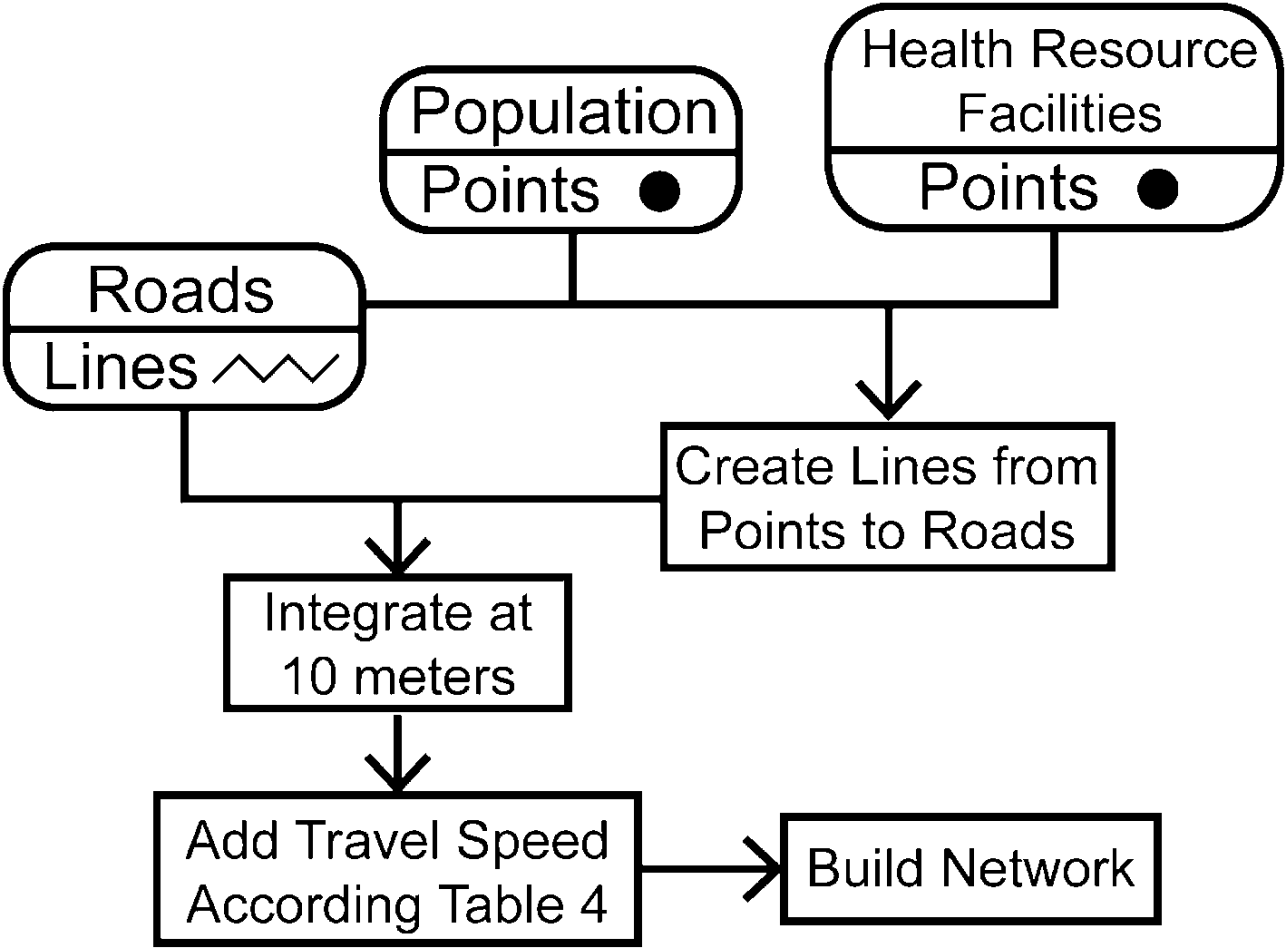
Steps to create network used to determine. First, *lines* are created between points and existing roads. Second, *lines* are integrated using a 10 m tolerance to ensure network connectivity. Finally travel time is calculated using estimate travel speed in Table 1 and the network is compiled

Figure 8 illustrates the steps to produce the road network. A GIS network is used to represent and study complex scenarios such as transportation networks. Networks are comprised of: edges (lines), which represent how entities move along the environment; junctions (points), which dictate how entities travel from line to line; and turns, which are optional elements limiting the movement at junctions between edges.

It was necessary to ensure that the point locations for health facilities and population centers fell on the network, otherwise travel between points could not be determined. An additional 2331 road segments were created as straight lines from the populated place and health resource facilities points to the nearest OSM road segment. These connector roads accounted for 738.3 miles of new roads, with an average length of 0.32 miles and longest length of 6.3 miles.

OSM data is not provided in a fully developed network format. To ensure connectivity between all elements, the Network Analyst Integrate Tool was used to snap together all roads segments within 10 m. No turns were included in the network, meaning no cost was added to the routes generated from moving to one element to another and that it is possible to turn in any direction at any junction. The small-scale alterations to the road network dataset were designed to create a functional network and are expected to have little effect on the results.

#### Quantifying health care access

Once a network has been created it is possible to use the closest facility tools of the ArcGIS Network Analyst extension to determine the fastest route between pairs of origins and destinations. Total health care access can then be determined as the summation of travel times, weighted by population, from (a) each populated place to diagnosis, and (b) each point of diagnosis to care.

Current health care access was determined by calculating the total shortest distances between populated places, health resource facilities indicated to have POCT from the Kost et al. study [15], and Srinagarind hospital in Khon Kaen.

Exploring health care access under widespread integration strategies involved two analyses to evaluate how implementing POCT at different administrative levels will affect health care access. These simulate situations in which the health network adopted policies that dictated certain facilities had to implement POCT. Two scales of integration were used. The first quantified health care access if POCT existed in every hospital. The second analysis quantified health care access if POCT existed in every health resource facility (hospitals and health promoting hospitals).

The resource limited strategy simulates if a health network only has limited resources to improve their health networks. By using Network Analyst’s location-allocation analysis with population as a weight, it is possible to determine the best locations (i.e. shortest total travel distance) given a certain set of constraints. Five analyses were performed which evaluated different resource limited implementation strategies:

1. Rearranging existing POCT to optimal locations.
2. Optimizing placement of 5 additional POCT while keeping the existing resources where they are.
3. Optimizing placement of 10 additional POCT while keeping the existing resources where they are.
4. Optimizing placement of 5 additional POCT while allowing the existing resources to be rearranged.
5. Optimizing placement of 10 additional POCT while allowing the existing resources to be rearranged.

## Abbreviations

POCT: point-of-care testing
GIS: geographic information systems
SWN: small-world network
ASSURED: affordable, sensitive, specific, user-friendly, rapid/robust, equipment-free, and deliverable
SCP: spatial care path™
OSM: OpenStreetMap
